# Complementary Rhizosphere Microbial Strategies Drive Functional Specialization in Coastal Halophyte Succession: Differential Adaptation of *Suaeda glauca* and *Phragmites communis* to Saline–Alkali Stress

**DOI:** 10.3390/microorganisms13061399

**Published:** 2025-06-16

**Authors:** Hao Dai, Mingyun Jia, Jianhui Xue, Zhuangzhuang Liu, Dongqin Zhou, Zhaoqi Hou, Jinping Yu, Shipeng Lu

**Affiliations:** 1 Institute of Botany, Jiangsu Province and Chinese Academy of Sciences, Nanjing 210014, China; hdai@njfu.edu.cn (H.D.); jiamingyun@jib.ac.cn (M.J.); jhxue@njfu.edu.cn (J.X.); zzliu@jib.ac.cn (Z.L.); zhoudongqin@cnbg.net (D.Z.); houzhaoqi939@sina.com (Z.H.); yujinping@cnbg.net (J.Y.); 2 College of Ecology and Environment, Nanjing Forestry University, Nanjing 210037, China; 3 Jiangsu Key Laboratory for the Research and Utilization of Plant Resources, Nanjing 210014, China; 4 Nanjing Botanical Garden Mem. Sun Yat-Sen, Nanjing 210014, China

**Keywords:** *Suaeda glauca*, *Phragmites communis*, rhizosphere microbiome, saline–alkali soil, functional specialization, halophyte adaptation, microbial succession

## Abstract

While rhizosphere microbiome functions in saline soils are well documented, complementary microbial strategies between pioneer and late-successional halophytes remain unexplored. Here, we used 16S rRNA sequencing and FAPROTAX functional prediction to compare the rhizosphere bacterial communities of two key halophytes—*Suaeda glauca* and *Phragmites communis*—in a reclaimed coastal wetland. The results demonstrate that both plants significantly restructured microbial communities through convergent enrichment of stress-tolerant taxa (*Firmicutes*, *Pseudomonas*, *Bacillus*, and *Planococcus*) while suppressing sulfur-oxidizing bacteria (*Sulfurovum* and *Thiobacillus*). However, they exhibited distinct microbial specialization: *S. glauca* uniquely enriched organic-matter-degrading taxa (*Promicromonospora* and *Zhihengliuella*) and upregulated aromatic compound degradation (2.29%) and ureolysis (0.86%) according to FAPROTAX analysis, facilitating carbon mobilization in early successional stages. Notably, *P. communis* selectively recruited nitrogen-cycling *Serratia*, with increased nitrate respiration (3.51% in *P. communis* vs. 0.91% in *S. glauca*) function, reflecting its higher nitrogen demand. Environmental factors also diverged: *S. glauca*’s microbiome correlated with potassium and sodium, whereas *P. communis* responded to phosphorus and chloride. These findings uncover distinct microbial recruitment strategies by halophytes to combat saline stress—*S. glauca*–*P. communis* synergy through microbial carbon-nitrogen coupling—offering a template for consortia design in saline soil restoration.

## 1. Introduction

Coastal wetlands are among Earth’s most productive ecosystems, delivering essential services such as carbon sequestration, shoreline stabilization, and biodiversity support [1,2]. However, these fragile ecosystems face escalating threats from anthropogenic pressures, notably large-scale coastal reclamation projects that disrupt hydrological regimes and sediment dynamics [3]. Among the ecological processes underpinning wetland resilience, the rhizosphere microbiome plays a critical role in sustaining soil formation, nutrient cycling, and plant establishment, especially in saline, disturbed landscapes like reclaimed coasts. This highlights the urgent need to understand microbial community dynamics under engineered conditions. The Linhong Estuary in Jiangsu Province, China, exemplifies such anthropogenic transformation, experiencing substantial ecological modifications after a major coastal reclamation project in 2020 [4,5]. This artificial landscape transformation created a quasi-experimental setting to investigate primary succession in neo-formed coastal wetlands. Critically, unlike centennial-scale natural succession, these engineered systems demand immediate microbial community assembly to initiate ecosystem functioning—a process where pioneer plants play pivotal roles [6]. Recent breakthroughs reveal that such pioneer species function as biogenic engineers, enabling rapid soil conditioning through rhizosphere-mediated niche construction [7].

As typical representatives of contrasting successional strategies, the halophyte *Suaeda glauca* (SG, early-successional pioneer) and *Phragmites communis* (PC, late-successional dominant) have concurrently colonized the Linhong Estuary’s reclaimed area [4,5,8,9]. This unique co-occurrence, which is rare in natural wetlands, establishes a model system to investigate how divergent plant functional traits mediate pedogenesis and microbiome assembly during anthropogenic wetland formation, particularly under the compressed timescales imposed by coastal reclamation.

The newly formed soils in the Linhong Estuary’s reclaimed area exhibit distinct physicochemical properties—notably a high clay content, elevated salinity, and compacted structure—that contrast sharply with natural wetland soils. These dredged marine sediments undergo accelerated pedogenesis driven by plant–soil–microbe interactions, where the co-occurring pioneer species *S. glauca* and *P. communis* may serve as key ecological engineers through differential root exudation, litter quality, and microbiome recruitment, as previous studies have revealed [5,10]. Yet, despite recognizing vegetation’s overarching role, the species-specific mechanisms governing microbial community assembly and functional adaptation in these anthropogenically forced systems remain a critical knowledge gap, particularly given the compressed ecological timescales imposed by coastal reclamation.

In this study, we aimed to investigate how plant-driven rhizosphere microbiome assembly contributes to ecosystem development in saline reclaimed wetlands, using the Linhong Estuary in Jiangsu Province, China, as a case study. Specifically, we address three key research objectives: (1) to identify species-specific differences in rhizosphere bacterial community composition between these successional strategists; (2) to decipher functional disparities within their respective rhizosphere microbiomes; and (3) to determine the edaphic drivers underlying microbial community differentiation. These objectives collectively advance our understanding of plant-mediated ecosystem development in rapidly constructed coastal wetlands.

## 2. Materials and Methods

### 2.1. Study Region

**Figure 1:**
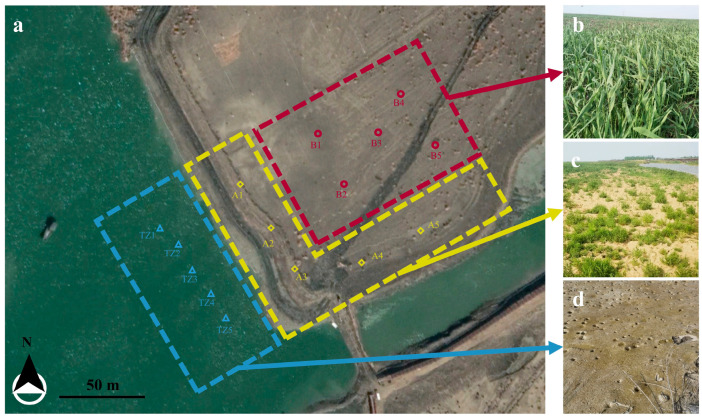
Information about the sampling site. (**a**) Newly designated coastal wetland zone at Linhong Estuary in Jiangsu Province, China. (**b**) *Phragmites communis* dominating area (sample B1 to B5); (**c**) *Suaeda glauca* dominating area (sample A1 to A5); (**d**) tidal zone area (sample TZ1 to TZ5).

The sampling area is situated within the newly designated coastal wetland zone—encompassing a 400 m diameter area (centered on coordinate 34°46′18.05″ N, 119°13′10.54″ E)—located at Linhong Estuary in Lianyun New Town, Lianyungang City, Jiangsu Province, China (Figure 1a). The newly formed coastal silt soils were formed as a result of the reclamation project, where original intertidal sediments were capped with dredged marine deposits. After two years of primary succession, distinct vegetation patterns emerged: (1) plot A adjacent to the tidal zone developed dominant *S. glauca* communities (Figure 1c), exhibiting characteristic pioneer colonization; (2) plot B (50 m inland) transitioned to *P. communis*-dominated stands (Figure 1b), with only scattered *S. glauca* individuals persisting under competitive suppression; and (3) the unvegetated tidal zone (Plot TZ, Figure 1d). This spatial zonation mirrors classic successional gradients, providing a chronosequence-like design to examine plant–microbe–soil interactions across successional stages.

### 2.2. Sample Collection

Sampling was conducted across the Linhong Estuary reclamation area on 20 May 2022. Five 50 cm × 50 cm quadrats were established in each vegetated plot (plot A dominated by *S. glauca* (SG) and plot B dominated by *P. communis* (PC)), with three sample types collected per quadrat: (1) rhizosphere soil was defined as the soil tightly adhering to the root surface after gently shaking off loosely attached soil. The entire plants were carefully uprooted, and the root systems were lightly shaken to remove loosely attached bulk soil. The remaining soil closely associated with the root surface was collected as the rhizosphere soil (marked as ‘rhizo’); (2) the non-rhizosphere soil located within 5 cm of the root zone at a depth of 5–15 cm (marked ‘soil’); and (3) bare soil controls from a depth of 5–15 cm for soil physicochemical analysis. In the unvegetated tidal zone (Plot TZ), only bare soil samples were collected. Whole plants of *S. glauca* and *P. communis* were carefully uprooted, with loose bulk soil gently shaken off roots before transferring root systems with adhering rhizosphere soil into sterile bags. Bulk soil samples were collected using ethanol-sterilized tools. All samples were immediately preserved on ice and subsequently stored at −80 °C until analysis. A total of five biological replicates per sample type (i.e., five ‘rhizo’ and five ‘soil’ samples for each plant species, and five bare soil samples from Plot TZ) were collected. For downstream analyses, all five biological replicates per group were used for DNA extraction and sequencing (‘rhizo’ and ‘soil’ samples from Plot SG, PC, and TZ), and soil physicochemical property analysis (bare soil controls from all plots). This standardized protocol ensured consistent collection of rhizosphere, bulk soil, and control samples across all study plots.

### 2.3. Soil Physicochemical Analysis

Soil pH was measured using a calibrated pH meter (PHS-3E, INESA, Shanghai, China) in a 1:5 (*w*/*v*) soil–water suspension. Electrical conductivity (EC) was determined with a digital conductivity meter (DDS-307, INESA, Shanghai, China) under identical suspension conditions. Soil total nitrogen (TN) was measured by Kjeldahl digestion and distillation, while total phosphorus (TP) was measured by the lithium fusion method. Flame photometry (FP6450, Shanghai INESA, Shanghai, China) was employed for total potassium (TK) determination. Available phosphorus (AP) was extracted using 0.5 M NaHCO_3_ (pH 8.5) and measured spectrophotometrically (UV-1800PC, MAPADA, Shanghai, China) at 700 nm via the molybdenum blue method. Exchangeable potassium (AK) was extracted with 1 M ammonium acetate (NH_4_OAc, pH 7.0) and quantified by flame photometry. Available nitrogen (AN) was assessed through the alkaline hydrolysis diffusion method. Soil organic carbon (SOC) was determined by the potassium dichromate (K_2_Cr_2_O_7_) wet oxidation method. Water-soluble cations K^+^, Ca^2+^, Na^+^, and Mg^2+^ (Avail. K, Avail. Ca, Avail. Na, Avail. Mg) were analyzed by flame atomic absorption spectroscopy (AA900T, PerkinElmer, Waltham, MA, USA). Water-soluble anion SO_4_^2−^ and Cl^−^ concentrations (Avail. SO_4_, Avail. Cl) were measured using ion chromatography (ICS-900, Thermo Fisher Dionex, Sunnyvale, CA, USA). Water-soluble anion HCO_3_^−^ (Avail. HCO_3_) was determined by acid–base titration with 0.01 M HCl using a bromocresol green-methyl red mixed indicator. Sodium adsorption ratio (SAR) was calculated using the following formula:

(1) $$\mathrm{SAR}\;=\frac{\mathrm{Avail}.\mathrm{Na}}{\sqrt{\left(\mathrm{Avail}.\mathrm{Ca}+\mathrm{Avail}.\mathrm{Mg}\right)/2}}$$

### 2.4. Plant Compartment Separation

Root–rhizosphere separation was conducted using a standardized sonication-based protocol. After manually removing loosely adhered bulk soil, root systems were transferred to 50 mL sterile centrifuge tubes containing epiphyte removal buffer (50 mM potassium phosphate buffer with 0.1% (*v*/*v*) Triton X-100). Mechanical homogenization was performed using a FastPrep-24 classic bead beating instrument (MP Biomedicals, Santa Ana, CA, USA) at a frequency of 4 m/s for 5 s to ensure uniform tissue disruption. Subsequent sonication was carried out under controlled conditions (4 °C, 160 W output) with pulsed cycles of 30 s sonication followed by 30 s cooling intervals for a total duration of 10 min. Following sonication, roots were aseptically removed from suspension. The remaining solution containing rhizosphere material was centrifuged (8000× *g*, 5 min, 4 °C). The supernatant was carefully decanted and preserved at −20 °C for subsequent analysis, while the pelleted material was designated as the rhizosphere compartment.

### 2.5. Extracellular DNA Removal

To selectively remove extracellular DNA (eDNA) before bulk sediment DNA extraction, we implemented a modified chemical treatment protocol [11]. For each sample, 2 g of sediment was subjected to a two-stage eDNA extraction process: Firstly, samples were incubated with 2 mL of carbonate dissolution solution (0.43 M sodium acetate, 0.43 M acetic acid, 10 mM EDTA, 100 mM sodium metaphosphate, 3% (*w*/*v*) NaCl, pH 4.7) for 1 h at room temperature (25 °C ± 1 °C) with constant orbital shaking (300 rpm). The slurry was subsequently treated with 16 mL of alkaline lysis buffer (300 mM Tris-HCl, 10 mM EDTA, 3% NaCl, pH 10.0) under identical agitation conditions for an additional hour. Following incubation, samples were centrifuged (10,000× *g*, 20 min, 25 °C) to pellet the eDNA-depleted sediment. The resulting pellet, devoid of dissolved and sediment-adsorbed extracellular DNA, was used for subsequent prokaryotic DNA extraction and community analysis.

### 2.6. DNA Extraction

DNA was extracted from 0.6 g of soil combined with 0.7 g of silica beads (0.1 mm in diameter) and 750 μL of 120 mM NaPO_4_ buffer (pH 8) in a 2 mL spiral cap tube, followed by the addition of 250 μL TNS (500 mM Tris-HCl pH 8.0, 100 mM NaCl, 10% SDS (*w*/*v*) [12]. Mechanical lysis was performed twice using the FastPrep-24 bead beating instrument at a frequency of 6.5 m/s for 45 s. The homogenate was centrifuged (20,817× *g*, 20 min, 4 °C), and the supernatant was transferred to a fresh 2 mL tube. Sequential purification steps included (1) adding one volume of phenol–chloroform–isoamyl alcohol (25:24:1), vortexing, and centrifuging (20,817× *g*, 5 min, 4 °C); (2) repeating the process with chloroform–isoamyl alcohol (24:1); and (3) precipitating nucleic acids with two volumes of PEG solution (20,817× *g*, 90 min, 4 °C). The pellet was washed with 500 μL of ice-cold 70% ethanol (20,817× *g*, 30 min, 4 °C), air-dried, and resuspended in 50 μL EB buffer. DNA concentration was quantified via NanoDrop (NanoDrop One, Thermo Fisher Scientific, Wilmington, DE, USA) and stored at −80 °C.

### 2.7. Library Construction, Sequencing, and Statistical Analysis

For the bacterial community, nearly the full length of bacterial 16S rRNA genes were amplified using primer set 27F/1492R, purified (AMPure^®^ PB beads, PacBio, Menlo Park, CA, USA), and quantified (Qubit 4.0). Equimolar pooled amplicons were processed into SMRTbell libraries (Prep Kit 3.0) and sequenced on a PacBio Sequel IIe system (Majorbio, Shanghai, China) to generate HiFi reads via circular consensus sequencing (SMRT Link v11.0). Subsequently, DADA2 in the QIIME2 pipeline was used for ASV calling and SILVA v138 for taxonomy classification. Bioinformatic analysis of the soil microbiota was carried out using the Majorbio Cloud platform (https://cloud.majorbio.com, accessed on 11 March 2024). Based on the ASV information, alpha diversity indices, including Ace, Chao1, Shannon, and coverage index, were calculated with Mothur v1.30.1. Data visualization was performed with R (version 3.2.3). Analyses included Venn-diagram generation, stacked bar plots of community composition, principal coordinate analysis (PCoA), Kruskal–Wallis multiple-group comparisons, and distance-based redundancy analysis (db-RDA). PCoA was chosen over NMDS to visualize beta diversity patterns because it effectively summarizes complex community composition data in a reduced-dimensional space, facilitating comparisons across samples. Similarly, db-RDA was selected to statistically quantify the relationship between community composition and measured soil variables, providing a constrained ordination approach that directly links environmental drivers to microbial patterns. The bacterial function was predicted using FAPROTAX_1.2.1 software.

Soil physicochemical data were analyzed in SPSS 22.0, and further descriptive, variance, and Pearson correlation analyses were performed.

## 3. Results

### 3.1. Analysis of Soil Physicochemical Properties

Soil physicochemical index determination results are shown in Table 1. The results indicate that soil pH across all study areas remained strongly alkaline (8.33–8.43), consistent with typical coastal saline–alkaline soils. Electrical conductivity (EC) was significantly higher in plot A (dominated by *Suaeda glauca*) than in TZ (tidal zone) and plot B (dominated by *Phragmites communis*), suggesting greater salt accumulation in *S. glauca* soils. Soluble ion analysis revealed that available Na^+^ and Cl^−^ concentrations in plot A were significantly elevated compared to TZ and plot B, confirming *S. glauca*’s adaptation to hypersaline conditions. Although TZ showed lower EC than plot A, its available HCO_3_^−^ and SO_4_^2−^ contents were significantly higher than vegetated areas, indicating sulfate and bicarbonate dominance in tidal zones versus potential plant-mediated ion transformation in vegetated zones. The sodium adsorption ratio (SAR) in both vegetated areas (348.80 in plot A; 315.08 in plot B) significantly exceeded TZ values, aligning with their elevated soluble sodium levels.

Nutrient composition exhibited contrasting trends: TZ contained significantly higher TN, AN, TP, and SOC than vegetated areas, likely due to marine-derived nutrient inputs and reduced plant uptake. AP showed no inter-area differences, suggesting phosphorus availability was uniformly constrained by alkaline conditions. TK was significantly higher in TZ and plot B than plot A, while AK showed the opposite pattern (*S. glauca* and *P. communis* areas > TZ), implying that vegetation promotes potassium mobilization. Pearson analysis revealed significant negative correlations between salinity indicators (EC, Na^+^, and Cl^−^) and fertility parameters (TP, SOC, AN, TN, and TK) (Figure S1), demonstrating salinity’s suppressive effect on soil fertility.

In summary, TZ displayed higher nutrient loads (TN, TP, and SOC) despite lower EC, while *S. glauca*-dominated soils showed extreme salinity adaptation (high EC, Na^+^, and Cl^−^). *P. communis* areas exhibited intermediate salinity-nutrient characteristics with elevated TK and AK, suggesting active desalination and nutrient cycling processes.

### 3.2. Analysis of Bacterial Community Diversity

Alpha diversity represents the diversity of each sample within the habitat. In this study, no significant differences were observed in α-diversity indices (ACE, Chao1, Shannon, and Coverage) between the TZ and bare soils of vegetated areas (plots A and B) (Figure S2a–d), suggesting that bacterial community richness and diversity remain relatively stable in unvegetated soils despite variations in environmental parameters and salinity distribution.

The regulation of rhizosphere microbial diversity by *S. glauca* showed environment-dependent patterns (Figure S2e–h). In plot A, no significant differences occurred in any α-diversity indices between the *S. glauca* rhizosphere and bare soil. In plot B, *S. glauca* rhizosphere significantly reduced ACE and Chao1 indices (*p* < 0.05) while increasing coverage values, with no significant impact on the Shannon index. *P. communis* exhibited more pronounced rhizosphere selection effects (Figure S2i–l). In both plots A and B, *P. communis* significantly decreased ACE, Chao1, and Shannon indices (*p* < 0.01) while increasing coverage values, indicating strong root-mediated selection that inhibited most bacterial taxa while enriching specific groups.

**Figure 2:**
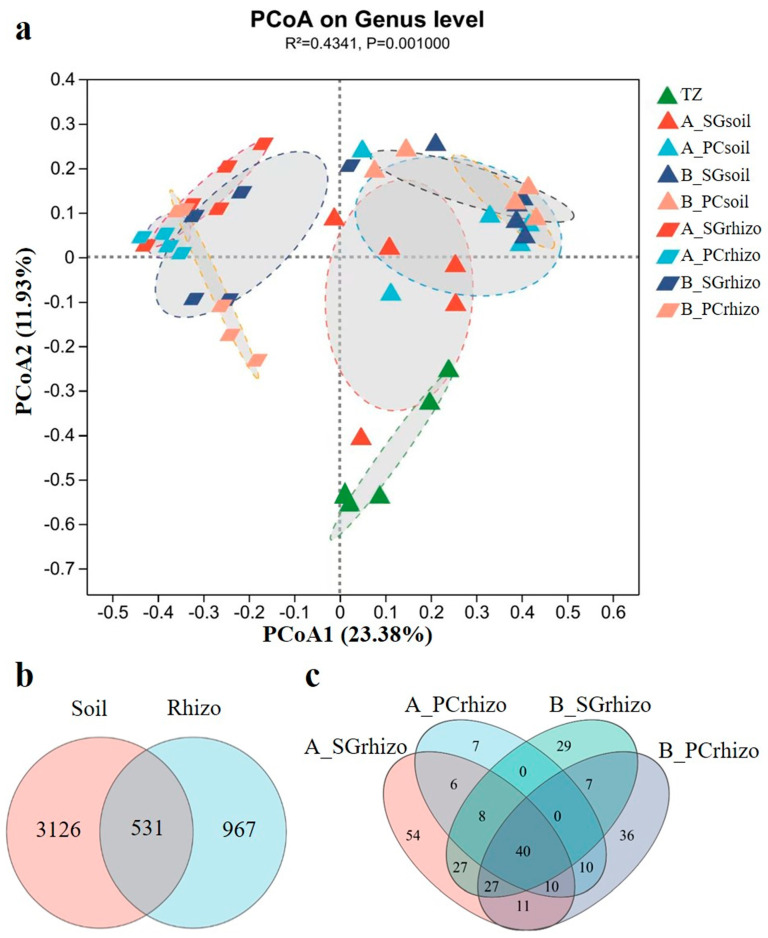
(**a**) Principal coordinate analysis (PCoA) of bacterial communities on the genus level of all samples. Symbols with the same shape and color indicate biological replicates. Cycles indicate different group ellipses. (**b**) Venn diagrams showing the number of shared and unique bacterial ASVs in all rhizosphere and peri-root soil samples. (**c**) Venn diagrams showing the number of shared and unique bacterial genera in the rhizosphere and peri-root soil samples of plot A and plot B. TZ, tidal zone; A, *Suaeda glauca*-dominant area; B, *Phragmites communis*-dominant area; SG, *Suaeda glauca*; PC, *Phragmites communis*; soil, peri-root soil sample; rhizo, rhizosphere sample.

The PCoA analysis revealed significant differences in bacterial community composition among different sample types (TZ, peri-soil, and rhizo), with clear spatial separation observed in the ordination plot (Figure 2a). Notably, A_SUrhizo and B_SUrhizo samples exhibited adjacent clustering patterns, indicating similarity in their bacterial community structure and composition. This trend was also observed in *P. communis* (PCrhizo)-associated communities (Figure 2a and Figure S3).

The Venn diagram analysis demonstrated that only 11.5% (531/4624) of total ASVs were shared between peri-soil and rhizosphere samples (Figure 2b), highlighting the strong selection pressure exerted by plants on their associated microbiota. Additionally, while 44.1% (171/388) of bacterial genera were commonly shared between *S. glauca* and *P. communis* rhizospheres (Figure 2c). This indicates that although these halophytes select for similar microbial taxa at higher taxonomic levels, substantial differences exist at finer phylogenetic resolution.

### 3.3. Analysis of Bacterial Community Composition

**Figure 3:**
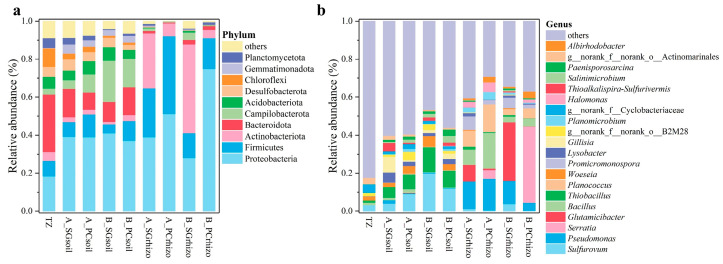
(**a**) Relative abundances of the dominant top 10 phyla in all soil samples. (**b**) Abundances of the dominant top 20 genera in all soil samples. TZ, tidal zone; A, *Suaeda glauca*-dominant area; B, *Phragmites communis*-dominant area; SG, *Suaeda glauca*; PC, *Phragmites communis*; soil, peri-root soil sample; rhizo, rhizosphere sample.

The relative abundance distribution of soil bacterial communities at both phyla and genus levels was analyzed, along with statistically significant differences (Figure 3). At the phylum level, the ten most abundant phyla were Proteobacteria, Firmicutes, Actinobacteriota, Bacteroidota, Campilobacterota, Acidobacteriota, Desulfobacterota, Chloroflexi, Gemmatimonadota, and Planctomycetota (Figure 3a). Comparative analysis revealed that bare soils in plots A and B showed significantly increased abundances of Proteobacteria, Campilobacterota, and Gemmatimonadota but decreased abundances of Actinobacteriota, Bacteroidota, Chloroflexi, and Planctomycetota relative to TZ (Figure S4). These patterns suggest that environmental conditions and physicochemical properties in bare soils selectively favor certain bacterial phyla while suppressing others. Vegetated soils in both plots A and B demonstrated distinct phylum-level patterns, with increased abundances of Proteobacteria, Firmicutes, and Actinobacteriota compared to bare soils, accompanied by decreases in other phyla.

At the genus level (Figure 3b), among the twenty most abundant genera, *Sulfurovum*, *Pseudomonas*, *Serratia*, *Glutamicibacter*, *Bacillus*, *Thiobacillus*, *Planococcus*, *Woeseia*, *Promicromonospora*, *Lysobacter*, and *Gillisia* were predominant.

Notably, bare soils in plots A and B exhibited significantly higher abundances of *Sulfurovum* and *Thiobacillus* but lower *Gillisia* abundance compared to TZ, indicating preferential proliferation of salt–alkali-tolerant or metabolically specialized bacteria in unvegetated environments. The presence of vegetation significantly increased the relative abundances of *Pseudomonas*, *Serratia*, *Bacillus*, *Planococcus*, and *Promicromonospora* while decreasing *Sulfurovum*, *Thiobacillus*, *Woeseia*, and *Gillisia* compared to bare soils (Figure S4).

These findings indicate that vegetation significantly restructures microbial communities through rhizosphere effects. The observed shifts likely result from plant-mediated changes in microenvironments and organic compound exudation, which create favorable conditions for specific bacterial groups while inhibiting others. In summary, both bare and vegetated soils exhibited distinct, habitat-specific patterns of bacterial community composition at multiple taxonomic levels.

**Figure 4:**
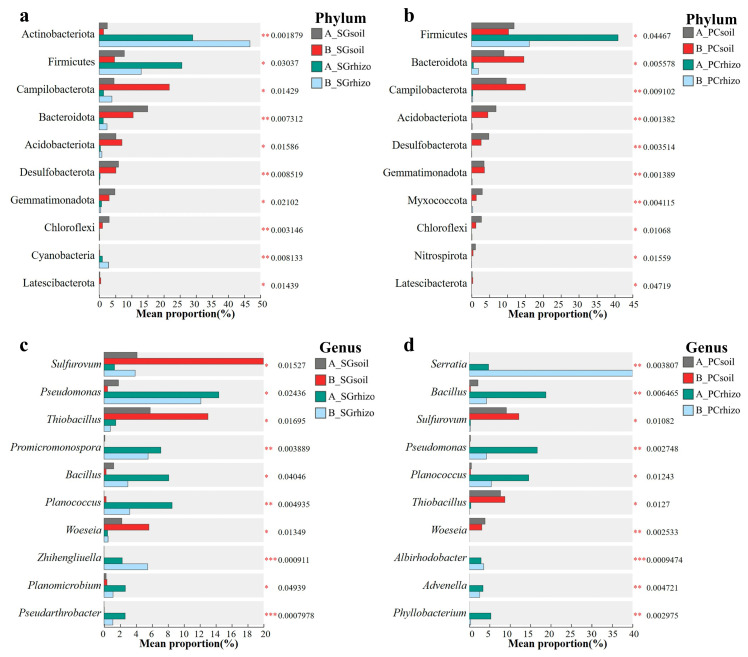
Differential bacterial community relative abundance between rhizosphere and peri-root soils of (**a**,**c**) *Suaeda glauca* and (**b**,**d**) *Phragmites communis*-dominating plots at (**a**,**b**) phylum and (**c**,**d**) genus levels (Kruskal–Wallis test), * 0.01 < *p* ≤ 0.05, ** 0.001 < *p* ≤ 0.01, *** *p* ≤ 0.001.

The results in Figure 4 demonstrate significant rhizosphere enrichment effects of bacterial communities in both plots A and B. In *S. glauca* rhizosphere soils, we observed substantial increases in the relative abundances of Actinobacteriota, Firmicutes, and Cyanobacteria at the phylum level (Figure 4a). Genus-level analysis revealed marked enrichment of *Pseudomonas*, *Promicromonospora*, *Bacillus*, *Planococcus*, *Zhihengliuella*, and *Pseudarthrobacter* (Figure 4c). In contrast, *P. communis* rhizosphere showed distinct selection patterns, with Firmicutes demonstrating significant phylum level enrichment in all samples (Figure 4b). However, multiple genera, including *Serratia*, *Bacillus*, *Pseudomonas*, *Planococcus*, *Albirhodobacter*, *Advenella*, and *Phyllobacterium* were significantly enriched (Figure 4d), confirming strong species-specific rhizosphere effects. The co-enrichment of *Bacillus*, *Pseudomonas*, and *Planococcus* in both rhizospheres suggests their fundamental roles in plant–microbe interactions within saline–alkaline ecosystems. Notably, both vegetation types significantly reduced abundances of *Sulfurovum*, *Thiobacillus*, and *Woeseia*, indicating potential vegetation-mediated regulation of sulfur cycling pathways to mitigate sulfide toxicity. These findings collectively demonstrate that different halophyte species exert distinct but equally significant influences on soil bacterial community structure through species-specific rhizosphere effects.

### 3.4. Analysis of Bacterial Community Composition and Environmental Factors

**Figure 5:**
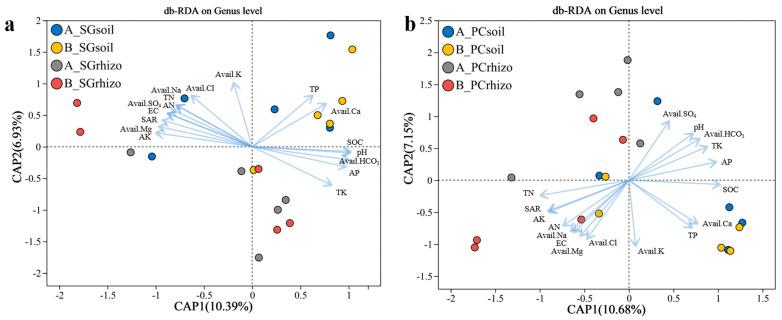
The db-RDA analysis of bacterial community composition and environmental factors of rhizosphere and peri-root soils of (**a**) *Suaeda glauca* and (**b**) *Phragmites communis*-dominating plots.

The distance-based redundancy analysis (db-RDA) showed the differential effects of environmental factors on bacterial community structures in *S. glauca* and *P. communis* rhizospheres. The results reveal distinct environmental response patterns between the two vegetation types, reflecting species-specific adaptations to saline–alkaline conditions (Figure 5 and Table 2). For *S. glauca*, total potassium (TK) showed the strongest correlation (*r*^2^ = 0.706, *p* = 0.001), suggesting K^+^ selective uptake as a key mechanism for Na^+^ toxicity mitigation. Significant associations with electrical conductivity (EC; *r*^2^ = 0.420, *p* = 0.013) and sodium adsorption ratio (SAR; *r*^2^ = 0.549, *p* = 0.004) further confirmed salinity and sodium accumulation as primary drivers of microbial community assembly. The absence of significant correlations with nutrient parameters (TP, TN, AP, AN, and SOC) implies microbial selection in *S. glauca* rhizospheres depends more on ionic homeostasis than nutrient availability. *P. communis* exhibited more complex environmental regulation, maintaining strong correlations with TK (*r*^2^ = 0.626, *p* = 0.002), EC (*r*^2^ = 0.668, *p* = 0.001), and SAR (*r*^2^ = 0.573, *p* = 0.002). Notably, additional significant relationships emerged with total phosphorus (TP; *r*^2^ = 0.440, *p* = 0.008), available chloride (*r*^2^ = 0.469, *p* = 0.003), and sodium (*r*^2^ = 0.379, *p* = 0.023), demonstrating broader environmental integration in *P. communis* rhizospheres. While TK, EC, and SAR represent core environmental determinants for both species, *P. communis* displays enhanced regulatory complexity through additional phosphorus and soluble ion interactions. These findings highlight fundamental differences in how these halophytes structure their rhizosphere microbiomes under saline–alkaline stress.

### 3.5. Predictive Analysis of Bacterial Community Function

**Figure 6:**
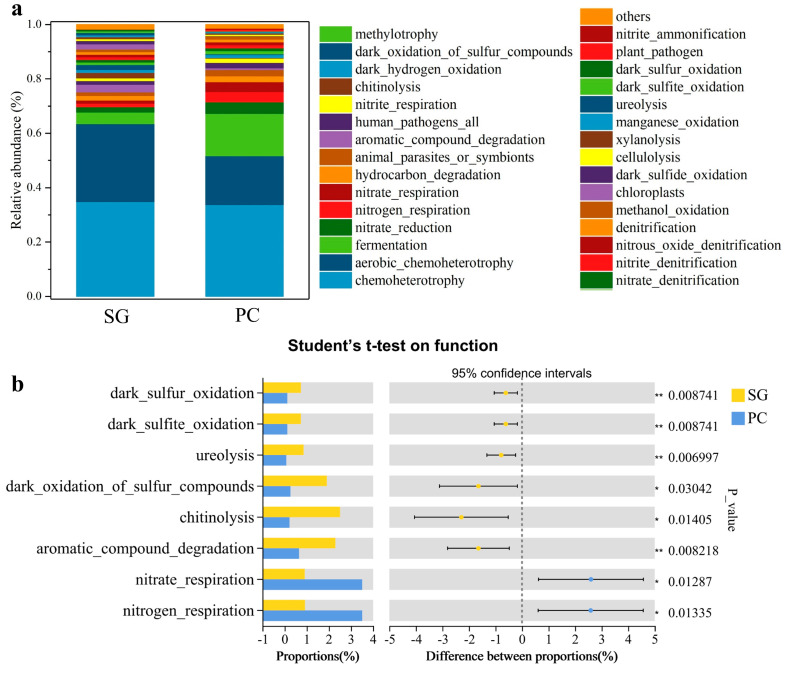
(**a**) Relative abundance histogram of top 30 bacterial community function prediction based on FAPROTAX for *Suaeda glauca* (SG) and *Phragmites communis* (PC) rhizosphere soil. (**b**) The difference in bacterial community function prediction between *Suaeda glauca* and *Phragmites communis* rhizosphere soil, * 0.01 < *p* ≤ 0.05, ** 0.001 < *p* ≤ 0.01.

The FAPROTAX database was employed to predict prokaryotic metabolic functions and ecologically relevant processes (e.g., nitrification, denitrification, and fermentation). Analysis revealed chemoheterotrophy, aerobic chemoheterotrophy, and fermentation as the dominant functions among the top 30 predicted bacterial functions in both *Suaeda glauca* and *Phragmites communis* rhizospheres. *S. glauca* rhizosphere soils showed significant enrichment of organic matter decomposition and sulfur cycling functions (Figure 6). Specifically, aromatic compound degradation, chitinolysis (2.1×), dark sulfite/sulfur oxidation (3.4×), and ureolysis (2.7×) were markedly elevated compared to *P. communis* samples, suggesting enhanced microbial capacity for recalcitrant organic decomposition and organic sulfur processing in *S. glauca* rhizospheres. In contrast, *P. communis* rhizospheres exhibited stronger nitrogen cycling potential, with nitrogen respiration (3.51% vs. 0.92%) and nitrate respiration (3.51% vs. 0.91%) showing 3.8-fold higher relative abundances than *S. glauca* rhizospheres. This demonstrates that *P. communis* preferentially enriches microorganisms capable of driving nitrate transformation and nitrogen gas release.

These findings reveal fundamental differences in functional potential between rhizosphere microbiomes: (1) *S. glauca* promotes organic matter decomposition and sulfur oxidation pathways, while (2) *P. communis* enhances nitrogen transformation processes. Such divergence highlights how plant-specific rhizosphere environments differentially shape microbial functional composition in coastal saline ecosystems.

## 4. Discussion

The succession of bacterial communities in coastal saline–alkali soils reflects the combined effects of environmental filtering and plant regulation. Bare soils (TZ and unvegetated areas A/B) maintained stable α-diversity (ACE, Chao, and Shannon) despite varying salinity conditions (Figure S2a–d), suggesting extreme saline–alkali stress selects for a conserved, stress-adapted core microbiome [13].

Plant establishment significantly altered bacterial community structure, with species-specific patterns. Specifically, *S. glauca* rhizospheres showed location-dependent effects, reducing richness (ACE/Chao1) in plot B while maintaining evenness (Shannon) (Figure S2), consistent with reported suppression of taxa expansion in coastal silt soils [5]. *P. communis* exhibited stronger selection, uniformly decreasing all α-diversity indices (Figure S2i–l), likely through root exudate-mediated suppression of generalists and enrichment of specialists [14]. Previous studies have shown that plant root exudates can regulate the composition and function of the rhizosphere microbial community and play an important role in shaping the rhizosphere microbial community [15,16,17].

β-diversity analysis confirmed vegetation-driven restructuring. Distinct microbial community clustering of TZ, bare soil, and rhizosphere samples was revealed by PCoA and limited ASV overlapping. Meanwhile, the rhizosphere samples in plots A and B clustered closely, suggesting convergent selection of microbial taxa by halophytes across salinity gradients. Low ASV-level similarity (19.2%) between *S. glauca* and *P. communis* despite genus-level convergence (44.1%) (Figure 2), aligning with reported species-specific microbial recruitment [18].

These patterns demonstrate that while bare soils maintain microbial diversity primarily through abiotic environmental filtering, vegetation establishment imposes strong biotic selection pressures that fundamentally reshape bacterial communities. The reduction in α-diversity observed particularly under *P. communis* reflects progressive niche specialization, likely due to selective root exudation strategies that promote beneficial taxa while suppressing less-adapted generalists. This form of microbial filtering may help *P. communis* stabilize its rhizosphere, promoting efficient nutrient cycling and improved resistance to salinity stress—both critical for survival in the compact, saline soils of reclaimed wetlands. For *S. glauca*, the more variable impact on richness but stable evenness suggests a different recruitment strategy—possibly favoring microbial taxa that contribute to early-stage soil conditioning without overly narrowing the microbial pool. This flexibility may enhance its adaptability to microhabitat variability, aiding its pioneer role during initial colonization. Concurrently, vegetation may drive β-diversity restructuring by creating species-specific chemical gradients that select distinct microbial assemblages. Ultimately, these plant-mediated selection processes shape functionally specialized rhizosphere microbiomes that perform critical ecosystem services in saline environments, including enhanced nutrient cycling and improved plant stress mitigation capabilities.

Comparative analysis revealed distinct patterns in bacterial community composition between unvegetated and rhizosphere soils. In bare soil samples, Proteobacteria dominated (37.15–41.07%), with elevated abundances of sulfur-oxidizing genera (*Sulfurovum* and *Thiobacillus*) (Figure 3) compared to TZ samples, consistent with previous findings in Yellow River Delta saline soils and other sites [17,19,20,21,22]. This suggests salt accumulation promotes chemolithotrophic bacteria capable of sulfur oxidation under high salinity.

Both *S. glauca* and *P. communis* rhizospheres exhibited an enrichment of microbial groups associated with plant-beneficial functions, including Firmicutes at the phylum level, and genera *Pseudomonas*, *Bacillus*, and *Planococcus* (Figure 4), suggesting potential selection for microbes that may contribute to stress mitigation. However, it is important to note that these genera comprise diverse species with varying ecological roles, including both beneficial and pathogenic strains. Those beneficial strains demonstrate well-documented plant-growth-promoting traits: phosphate solubilization, nitrogen cycling, and IAA production, et al. Xavier et al. reported that Firmicutes exhibit broad environmental adaptability and play important roles in plant stress resistance [23]. Similarly, specific strains within *Pseudomonas* and *Bacillus* have been widely recognized for their plant-growth-promoting functions under saline conditions, including phosphate solubilization, potassium release, and siderophore production [24,25]. Specifically, *Pseudomonas putida* strain TSAU1 enhances salt tolerance in soybean through root architecture modification that improves nitrogen/phosphorus uptake and nodulation [26]. In the mung bean, three Pseudomonas Mk strain mixed inoculation mitigates salt stress by regulating multiple physiological parameters, including photosynthesis and water relations [27]. *Bacillus cereus* or *B. subtilis* contributes to plant salt tolerance through several mechanisms: improving nitrogen cycling efficiency, stimulating osmolyte (proline and betaine) synthesis, inducing methionine through the ethylene pathway and ROS scavenging in tomato, and maintaining membrane stability in *Glycyrrhiza uralensis* [28,29,30]. Furthermore, *Bacillus paramycoides* application promotes wheat growth by enhancing soil phosphorus/nitrogen availability and modifying microbial community structure [31]. *Planococcus* sp. EK9 also demonstrate significant plant-growth-promoting potential under salinity, exhibiting multiple beneficial traits such as siderophore production, indole-3-acetic acid synthesis, nitrogen fixation, and phosphate solubilization [32]. For example, *Planococcus rifietoensis* improves *Beta vulgaris* performance under salt stress by enhancing germination, biomass accumulation, and photosynthetic efficiency while reducing ethylene production [33]. The inherent salt tolerance and glutathione production capacity of *Planococcus* strain ST3 and ST4 further support their effectiveness in saline environments [34]. Although functional verification at the species or strain level remains beyond the scope of this study, the significant enrichment of *Pseudomonas*, *Bacillus*, and *Planococcus* aligns consistently with the established literature documenting their roles in salt stress mitigation. This convergence of evidence underscores the promise of these microbial taxa in enhancing plant salinity resilience.

Notably, the rhizosphere soils of both *S. glauca* and *P. communis* exhibited significantly reduced abundances of sulfur-oxidizing bacteria, including *Sulfurovum*, *Thiobacillus*, and *Woeseia*. This observation demonstrates that vegetation exerts a dual regulatory effect on rhizosphere microbial communities: while actively enriching beneficial bacterial groups, it simultaneously suppresses potentially harmful microorganisms that may induce sulfide toxicity. The selective reduction of these sulfur-oxidizing bacteria likely alters sulfur cycling pathways in the rhizosphere, consequently decreasing the production of toxic sulfides and protecting plants from their damaging effects [20,21]. Notably, other halophytes like *Spartina alterniflora* employ distinct strategies for sulfur management, as reported in [22].

*S. glauca* shows specific enrichment of bacterial taxa, including *Promicromonospora*, *Zhihengliuella*, and *Pseudarthrobacter* in its rhizosphere (Figure 4a,c). These bacteria likely contribute to the plant’s salt tolerance through multiple mechanisms. *Promicromonospora* secretes gibberellic acid (GA), which has been shown to increase endogenous GA levels and stimulate growth in tomato plants [35]. *Zhihengliuella*, a known salt-tolerant bacterium, produces ACC deaminase [36] and has been demonstrated to improve antioxidant capacity in *Haloxylon aphyllum* by modulating various stress markers, including ascorbic acid, flavonoids, total phenols, proline, malondialdehyde, and catalase activity [37]. Additionally, under salt stress, *Zhihengliuella* treatment enhances chlorophyll a, iron, magnesium, manganese, and anthocyanin content while boosting catalase activity in *Seidlitzia rosmarinus*, thereby mitigating salt-induced damage [38]. *Pseudarthrobacter* also promotes plant growth under saline conditions; for example, *Pseudarthrobacter enclensis* inoculation improves maize growth and biomass by regulating osmotic balance, reducing sodium/potassium accumulation in plant tissues, and protecting the photosynthetic apparatus, resulting in increased chlorophyll a and b content [39]. These findings collectively reveal the microbial basis of *S. glauca*’s salt tolerance and provide a foundation for using halotolerant bacteria, either individually or in consortia, for saline–alkali soil remediation.

The rhizosphere of *P. communis* demonstrates significant enrichment of specific bacterial taxa, including *Serratia*, *Albirhodobacter*, *Phyllobacterium*, and *Advenella* (Figure 4b,d), which collectively support plant growth under saline–alkali stress conditions. Among these, *Serratia* has been well documented for its phosphate-solubilizing capacity, converting insoluble phosphates into plant-available forms through both solubilization and mineralization processes [40]. This mechanism has been shown to enhance phosphorus uptake and promote growth in crops including corn and soybean [41]. Beyond phosphorus mobilization, *Serratia* participates in nitrogen cycling through denitrification and nitrification pathways while also producing plant growth regulators such as indole-3-acetic acid and ACC deaminase [42,43,44]. The other enriched bacteria, though less extensively studied, exhibit distinct stress-alleviating properties. *Albirhodobacter*, as a halophilic bacterium, facilitates plant adaptation to saline environments [45]. *Phyllobacterium* demonstrates halotolerant characteristics that enhance plant tolerance to various abiotic stresses, including heavy metal toxicity and drought [46,47,48]. *Advenella* functions as a nitrite-denitrifying bacterium with organic matter decomposition capabilities, showing potential for bioremediation applications [49]. These findings collectively indicate that the bacterial taxa preferentially enriched in *P. communis* rhizosphere represent promising candidates for developing microbial inoculants to improve plant performance under abiotic stress conditions. The complementary functions of these bacteria in nutrient cycling, stress hormone regulation, and organic matter decomposition contribute to the observed resilience of *P. communis* in saline–alkali environments.

Environmental factor analysis demonstrated distinct regulatory patterns in the rhizosphere microbial communities of *S. glauca* and *P. communis*, reflecting their distinct physiological adaptations to saline–alkali conditions (Figure 5). In *S. glauca*-related samples, potassium (TK) emerged as the primary environmental determinant of microbial composition (*r*^2^ = 0.706, *p* = 0.001), indicating its potential role in potassium-selective uptake to counter sodium toxicity. Strong correlations were also observed with electrical conductivity (EC; *r*^2^ = 0.420, *p* = 0.013) and sodium adsorption ratio (SAR; *r*^2^ = 0.549, *p* = 0.004), confirming that salinity and sodium dynamics significantly influence microbial assembly in *S. glauca* rhizospheres. These findings support the hypothesis that S. *glauca* preferentially regulates ion homeostasis to maintain rhizosphere microbiome stability [50]. *P. communis* samples showed a more complex environmental regulation pattern, maintaining significant associations with TK (*r*^2^ = 0.626, *p* = 0.002), EC (*r*^2^ = 0.668, *p* = 0.001), and SAR (*r*^2^ = 0.573, *p* = 0.002) while also demonstrating additional correlations with total phosphorus (TP; *r*^2^ = 0.440, *p* = 0.008), available chloride (*r*^2^ = 0.469, *p* = 0.003), and available sodium (*r*^2^ = 0.379, *p* = 0.023). This broader environmental integration suggests *P. communis* employs a multifaceted strategy combining nutrient management and ion regulation to shape its rhizosphere microbiome.

These findings demonstrate that vegetation succession in saline–alkali soils drives significant and species-specific regulation of soil microbial communities through rhizosphere processes. Both S. *glauca* and *P. communis* selectively enriched distinct keystone microbial taxa that contribute to plant growth promotion and stress resistance. These microbial assemblages show specialized functional capacities in nutrient cycling (phosphorus, potassium, and nitrogen) and salt-tolerance mechanisms. These findings are consistent with previous research; for example, An et al. reported that the rhizosphere of *S. glauca* harbors a more functionally diverse and dominant bacterial community compared to that of *Tripolium vulgare* and that *S. glauca* promotes the turnover of key soil elements—carbon, nitrogen, sulfur, and iron—by enriching microbial taxa involved in these biogeochemical cycles [4]. Furthermore, our results reveal that vegetation actively modulates sulfur cycling pathways by suppressing potentially harmful sulfur-oxidizing bacteria (*Sulfurovum* and *Thiobacillus*), thereby mitigating sulfide toxicity in the rhizosphere environment.

These mechanistic insights establish both theoretical frameworks and practical approaches for saline–alkali soil remediation. The identification of functionally important, salt-tolerant microbial strains—whether applied individually or as designed consortia—provides tangible solutions for ecological restoration initiatives and sustainable agricultural practices in degraded coastal ecosystems. The study particularly highlights how native halophytes like *S. glauca* and *P. communis* can serve as models for developing microbiome-based land rehabilitation strategies.

Functional predictions of rhizosphere microbiomes in coastal saline–alkali soils demonstrate distinct metabolic strategies between *S. glauca* and *P. communis* under saline–alkali stress, reflecting vegetation-specific rhizosphere environments and regulatory mechanisms (Figure 6a). FAPROTAX analysis reveals that both vegetation types differentially regulate microbial functional modules to drive carbon and nitrogen cycling processes, creating complementary effects in saline–alkali soil restoration (Figure 6b).

The *S. glauca* rhizosphere shows significant enrichment of aromatic compound degradation (2.29%), chitinolysis (2.51%), and ureolysis (0.86%) functions. These findings suggest that *S. glauca* stimulates microbial decomposition of complex organic compounds through root exudates, potentially mobilizing fixed carbon sources (e.g., lignin from litter) to alleviate carbon limitation in barren environments [17]. This ‘carbon mining’ strategy aligns with *S. glauca*’s pioneer role, where microbially mediated organic matter turnover improves soil structure and facilitates the establishment of later-successional species like *P. communis* [4].

In contrast, the *P. communis* rhizosphere exhibits significantly higher nitrogen respiration (3.51%) and nitrate respiration (3.51%) compared to *S. glauca*, indicating preferential use of nitrate as an electron acceptor for denitrification [4]. While this process converts nitrate (NO_3_^−^) to gaseous nitrogen (N_2_O, N_2_), potentially exacerbating nitrogen limitation through nitrogen loss [51], *P. communis* may compensate through (1) deep root systems that activate mineral nitrogen [52], and (2) enrichment of *Serratia* (Figure 4d), which enhances nitrogen cycling to support the plant’s high productivity demands [42,43,44]. Meanwhile, *S. glauca*’s ureolysis enhancement suggests alternative ammonium (NH_4_^+^) acquisition through organic nitrogen mineralization [53], an advantageous strategy in tidally influenced habitats with high total nitrogen but low availability.

These results demonstrate vegetation-specific functional specialization through rhizosphere effects: *S. glauca* prioritizes carbon activation and organic matter decomposition, while *P. communis* emphasizes nitrogen cycling and supply management. This functional complementarity provides a theoretical framework for plant interactions in saline–alkali ecosystems and practical insights for microbial-based soil improvement strategies.

## 5. Conclusions

This study reveals how *Suaeda glauca* and *Phragmites communis* differentially regulate rhizosphere bacterial communities in coastal saline–alkali soils. While bare soils maintained stable α-diversity under extreme salinity, vegetation introduction drove species-specific microbial restructuring. Both plants enriched shared functional taxa (e.g., *Pseudomonas* and *Bacillus*) but exhibited distinct specialization: *S. glauca* selected for organic matter-degrading bacteria (*Promicromonospora* and *Zhihengliuella*) and suppressed sulfur oxidizers (*Sulfurovum* and *Thiobacillus*), aligning with its carbon-mining strategy to alleviate sulfide toxicity and improve soil fertility. In contrast, *P. communis* prioritized nitrogen-cycling genera (e.g., *Serratia*) and denitrification functions, supporting its high biomass demand despite potential nitrogen loss. Environmental drivers differed—*S. glauca*’s microbiome correlated strongly with potassium (TK) and sodium (SAR), whereas *P. communis* responded additionally to phosphorus (TP) and chloride (Avail. Cl), reflecting niche-specific adaptations.

These findings highlight complementary plant–microbe strategies: *S. glauca* enhances carbon mobilization for pioneer colonization, while *P. communis* optimizes nitrogen use for late-successional dominance. The suppression of harmful sulfur oxidizers by both plants underscores their role in mitigating sulfide stress. This work provides a mechanistic basis for using tailored microbial consortia (e.g., combining carbon-activating and nitrogen-cycling strains) to accelerate saline soil restoration. Future studies should validate these functional traits in situ and design synergistic inoculants to harness plant-specific microbial interactions for ecosystem rehabilitation.

## Figures and Tables

**Table 1:** Physicochemical properties of soil.

Properties	Sampling Sites		
	TZ	Plot A	Plot B
pH	8.33 ± 0.04 a	8.37 ± 0.08 a	8.43 ± 0.11 a
EC (mS/cm)	3.08 ± 0.15 b	4.31 ± 0.28 a	3.69 ± 0.56 b
TN (g/kg)	1.20 ± 0.03 a	1.06 ± 0.09 b	1.07 ± 0.03 b
TP (g/kg)	0.75 ± 0.01 a	0.70 ± 0.01 b	0.71 ± 0.02 b
TK (g/kg)	19.81 ± 0.90 a	18.13 ± 1.46 b	20.31 ± 0.41 a
AN (mg/kg)	73.03 ± 2.52 a	59.27 ± 9.89 b	57.04 ± 0.35 b
AP (mg/kg)	8.77 ± 1.21 a	8.60 ± 0.19 a	8.83 ± 0.26 a
AK (mg/kg)	438.40 ± 33.91 b	514.00 ± 40.03 a	502.20 ± 38.01 a
SOC (g/kg)	16.51 ± 0.92 a	11.70 ± 1.12 b	12.84 ± 0.44 b
Avail. K (mg/kg)	142.05 ± 14.38 a	156.85 ± 16.70 a	147.90 ± 15.17 a
Avail. Ca (mg/kg)	150.20 ± 23.01 a	103.30 ± 42.72 ab	84.85 ± 25.66 b
Avail. Na (mg/kg)	2730.00 ± 122.77 b	3780.50 ± 327.50 a	3200.00 ± 478.00 b
Avail. Mg (mg/kg)	156.55 ± 25.04 a	140.35 ± 38.63 a	125.80 ± 36.50 a
Avail. HCO_3_ (mg/kg)	42.58 ± 1.58 a	35.50 ± 2.30 b	36.60 ± 3.34 b
Avail. Cl (mg/kg)	3538.23 ± 136.52 b	5698.92 ± 753.95 a	4400.05 ± 743.97 b
Avail. SO_4_ (mg/kg)	1729.93 ± 170.86 a	1128.11 ± 247.87 b	1136.71 ± 194.57 b
SAR	220.45 ± 7.28 b	348.80 ± 29.73 a	315.08 ± 18.34 a

Note: The data in the table are average ± standard deviation; TZ, the soil samples from tidal zone area; plot A; the soil samples from *Suaeda glauca* dominating area; plot B, the soil samples from *Phragmites communis* dominating area; EC, electrical conductivity; TN, total nitrogen; TP, total phosphorus; TK, total potassium; AN, available nitrogen; AP, available phosphorus; AK, available potassium; SOC, organic carbon; Avail., available state; SAR, sodium adsorption ratio. Different lowercase letters of the same index in different samples indicate that there is a significant difference among the samples in this index (according to Duncan’s significance test, *p* < 0.05).

**Table 2:** Envfit environmental factor table for db-RDA analysis of *Suaeda glauca* and *Phragmites communis* rhizosphere soil.

Environmental Factors	*Suaeda glauca*		*Phragmites communis*	
	*r* ^2^	*p*	*r* ^2^	*p*
TK	0.7061	0.001 ***	0.6255	0.002 **
TP	0.2108	0.136	0.4399	0.008 **
TN	0.1138	0.369	0.0174	0.848
AP	0.1324	0.31	0.2356	0.112
AK	0.0392	0.727	0.1103	0.375
AN	0.2439	0.079	0.1117	0.338
SOC	0.0427	0.721	0.2755	0.053
Avail. HCO_3_	0.2924	0.065	0.2908	0.068
Avail. Cl	0.2163	0.141	0.4686	0.003 **
Avail. SO_4_	0.0117	0.91	0.0145	0.866
Avail. K	0.2127	0.126	0.114	0.378
Avail. Ca	0.1559	0.227	0.1562	0.234
Avail. Na	0.2613	0.09	0.3794	0.023 *
Avail. Mg	0.0368	0.699	0.089	0.46
pH	0.0794	0.502	0.2091	0.158
EC	0.4195	0.013 *	0.6676	0.001 ***
SAR	0.5492	0.004 **	0.5731	0.002 **

Note: The *r*^2^ value (coefficient of determination) quantifies the proportion of species distribution variance explained by each environmental factor, with smaller values indicating weaker influences; the *p*-value assesses the statistical significance of these correlations, where *p* < 0.05 indicates a significant relationship, * 0.01 < *p* ≤ 0.05, ** 0.001 < *p* ≤ 0.01, *** *p* ≤ 0.001.

## Data Availability

The original data presented in the study are openly available in China National Center for Bioinformation at CRA025859.

## Supplementary Materials

The following supporting information can be downloaded at https://www.mdpi.com/article/10.3390/microorganisms13061399/s1. Figure S1: Pearson correlation analysis of soil physicochemical properties; Figure S2: Bacteria α-diversity indices in peri-root soil and rhizosphere soil of different plants. (a–d) α-diversity indices in plots TZ, A, and B, (e–h) α-diversity indices in *Suaeda glauca* samples, (i–l) α-diversity indices in *Phragmites communis* samples. TZ, tidal zone; A, *S. glauca*-dominant area; B, *P. communis*-dominant area; SG, *S. glauca*; PC, *P. communis*; soil, peri-root soil sample; rhizo, rhizosphere sample; Figure S3: Principal coordinate analysis (PCoA) based on the relative abundance of bacterial genera in the different samples. (a) All rhizosphere soil and peri-root soil, (b) the rhizosphere soil and bare soil of *Suaeda glauca*, (c) the rhizosphere soil and peri-root soil of *Phragmites communis*, (d) the rhizosphere soil and peri-root soil of *S. glauca* in plot A, (e) the rhizosphere soil and peri-root soil of *S. glauca* in plot B, (f) the rhizosphere soil and peri-root soil of *P. communis* in plot A, (g) the rhizosphere soil and peri-root soil of *P. communis* in plot B; Figure S4: Relative abundance differences of bacterial communities in all soil samples based on the Kruskal–Wallis test: (a) phylum level and (b) genus level. TZ, tidal zone; A, *S. glauca*-dominant area; B, *P. communis*-dominant area; SG, *S. glauca*; PC, *P. communis*; soil, peri-root soil sample; rhizo, rhizosphere sample.

## Abbreviations

The following abbreviations are used in this manuscript:

TZ	tidal zone area
Plot A	*Suaeda glauca*-dominant area
Plot B	*Phragmites communis*-dominant area
SG	*Suaeda glauca*
PC	*Phragmites communis*
A_SGsoil	peri root soil of *Suaeda glauca* in plot A
A_PCsoil	peri root soil of *Phragmites communis* in plot A
A_SGrhizo	rhizosphere soil of *Suaeda glauca* in plot A
A_PCrhizo	rhizosphere soil of *Phragmites communis* in plot A
B_SGsoil	peri root soil of *Suaeda glauca* in plot B
B_PCsoil	peri root soil of *Phragmites communis* in plot B
B_SGrhizo	rhizosphere soil of *Suaeda glauca* in plot B
B_PCrhizo	rhizosphere soil of *Phragmites communis* in plot B
