# Supplementary material for: Complementary Rhizosphere Microbial Strategies Drive Functional Specialization in Coastal Halophyte Succession: Differential Adaptation of Suaeda glauca and Phragmites communis to Saline–Alkali Stress
Source: Microorganisms. 2025 Jun 16;13(6):1399. doi: 10.3390/microorganisms13061399 (PMC12195563; doi:10.3390/microorganisms13061399)
Supplement: Supplementary file 1 [file microorganisms-13-01399-s001.zip › Supplementary Material.pdf]

Article

Complementary Rhizosphere Microbial Strategies Drive Functional Specialization in Coastal Halophyte Succession: Differential Adaptation of *Suaeda glauca* and *Phragmites communis* to Saline–Alkali Stress

Hao Dai [1,2], Mingyun Jia [1,3,4], Jianhui Xue [1,2,3,4], Zhuangzhuang Liu [1,3,4], Dongqin Zhou [1,3,4], Zhaoqi Hou [1,3,4], Jinping Yu [1,3,4] and Shipeng Lu [1,3,4,*]

[1] Institute of Botany, Jiangsu Province and Chinese Academy of Sciences, Nanjing 210014, China; hdai@njfu.edu.cn (H.D.); jiamingyun@jib.ac.cn (M.J.); jhxue@njfu.edu.cn (J.X.); zzliu@jib.ac.cn (Z.L.); zhoudongqin@cnbg.net (D.Z.); houzhaoqi939@sina.com (Z.H.); yujinping@cnbg.net (J.Y.)

[2] College of Ecology and Environment, Nanjing Forestry University, Nanjing 210037, China

[3] Jiangsu Key Laboratory for the Research and Utilization of Plant Resources, Nanjing 210014, China

[4] Nanjing Botanical Garden Mem. Sun Yat-Sen, Nanjing 210014, China

[*] Correspondence: lvshipeng@jib.ac.cn; Tel.: +86-25-84347106

Figure S1 Pearson correlation analysis of soil physicochemical properties.

Figure S2 Bacteria α-diversity indices in peri-root soil and rhizosphere soil of different plants. (a-d) α-diversity indices in plots TZ, A, and B, (e-h) α-diversity indices in *Suaeda glauca* samples, (i-l) α-diversity indices in *Phragmites communis* samples. TZ, tidal zone; A, S. glauca dominant area; B, *P. communis* dominant area; SG, *S. glauca*; PC, *P. communis*; soil, peri-root soil sample; rhizo, rhizosphere sample.

Figure S3 Principal coordinate analysis (PCoA) based on the relative abundance of bacterial genera in the different samples. (a) all rhizosphere soil and peri-root soil, (b) the rhizosphere soil and bare soil of *Suaeda glauca*, (c) the rhizosphere soil and peri-root soil of *Phragmites communis*, (d) the rhizosphere soil and peri-root soil of *S. glauca* in plot A, (e) the rhizosphere soil and peri-root soil of *S. glauca* in plot B, (f) the rhizosphere soil and peri-root soil of *P. communis* in plot A, (g) the rhizosphere soil and peri-root soil of *P. communis* in plot B.

Figure S4 Relative abundance differences of bacterial communities in all soil samples based on the Kruskal–Wallis test, (a) phylum level, (b) genus level. TZ, tidal zone; A, *S. glauca* dominant area; B, *P. communis* dominant area; SG, *S. glauca*; PC, *P. communis*; soil, peri-root soil sample; rhizo, rhizosphere sample.

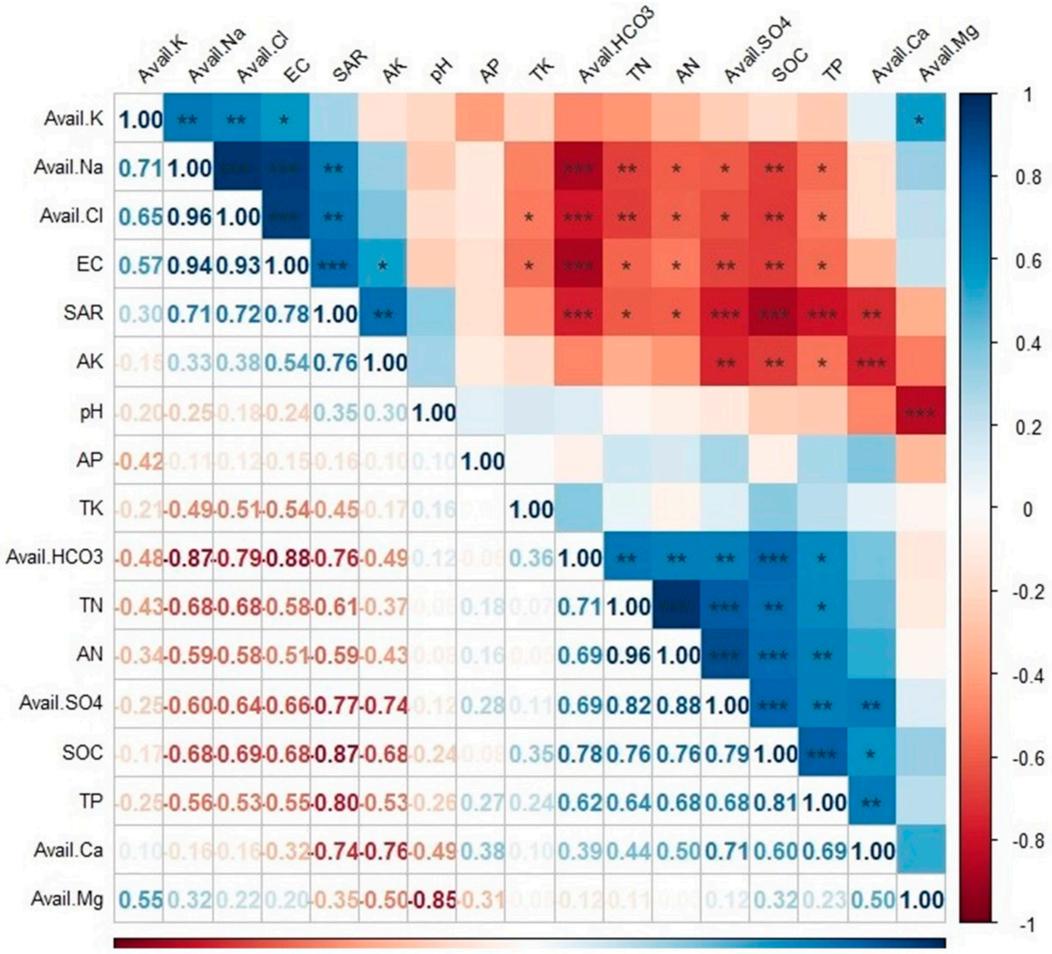

Figure S1 Pearson correlation analysis of soil physicochemical properties.

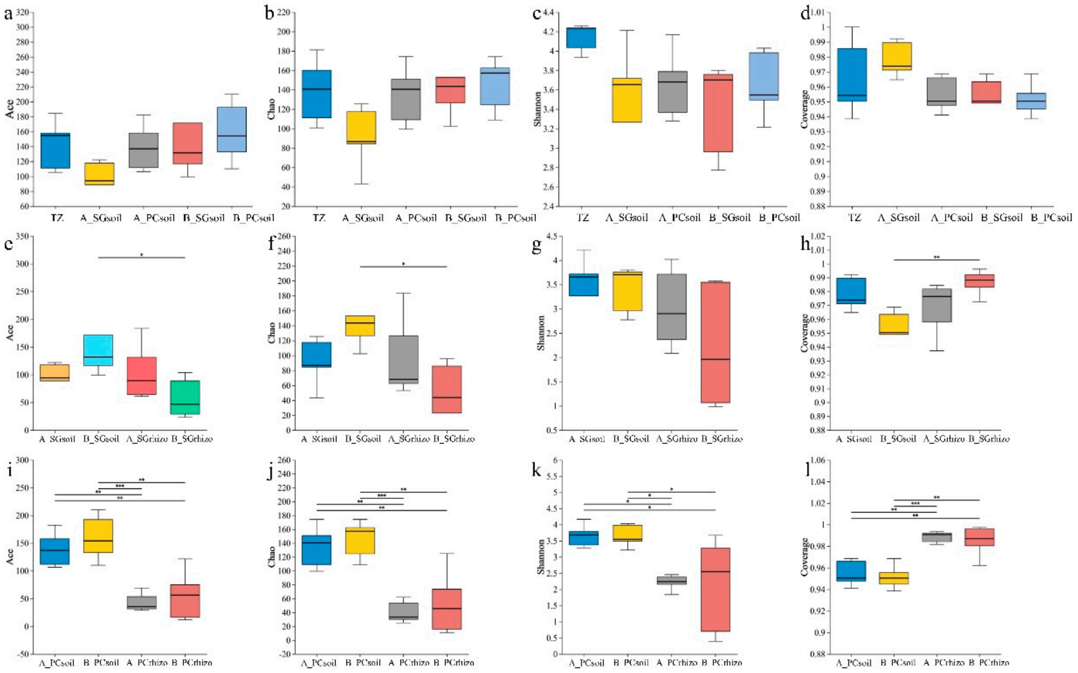

Figure S2 Bacteria α-diversity indices in peri-root soil and rhizosphere soil of different plants. (a-d) α-diversity indices in plots TZ, A, and B, (e-h) α-diversity indices in *Suaeda glauca* samples, (i-l) α-diversity indices in *Phragmites communis* samples. TZ, tidal zone; A, S. glauca dominant area; B, *P. communis* dominant area; SG, *S. glauca*; PC, *P. communis*; soil, peri-root soil sample; rhizo, rhizosphere sample.

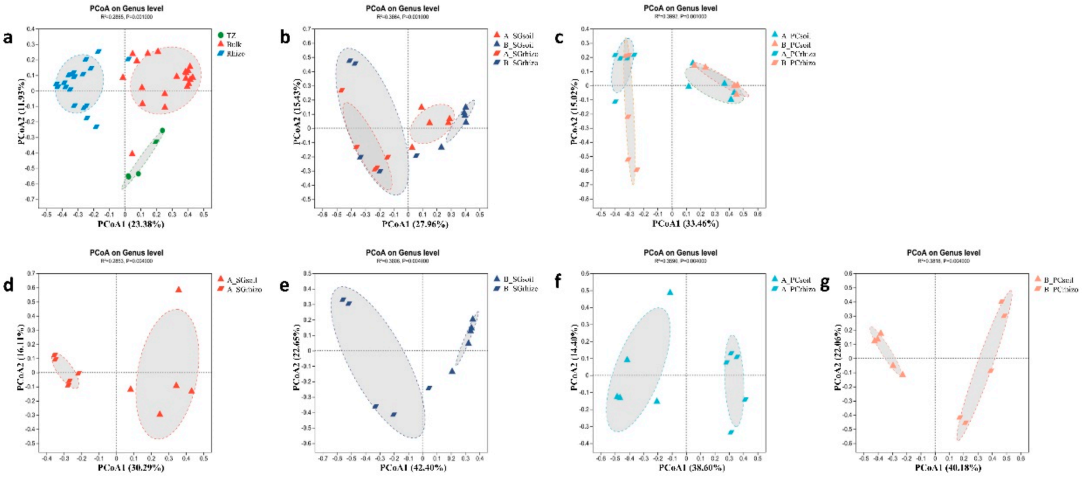

Figure S3 Principal coordinate analysis (PCoA) based on the relative abundance of bacterial genera in the different samples. (a) all rhizosphere soil and peri-root soil, (b) the rhizosphere soil and bare soil of *Suaeda glauca*, (c) the rhizosphere soil and peri-root soil of *Phragmites communis*, (d) the rhizosphere soil and peri-root soil of *S. glauca* in plot A, (e) the rhizosphere soil and peri-root soil of *S. glauca* in plot B, (f) the rhizosphere soil and peri-root soil of *P. communis* in plot A, (g) the rhizosphere soil and peri-root soil of *P. communis* in plot B.

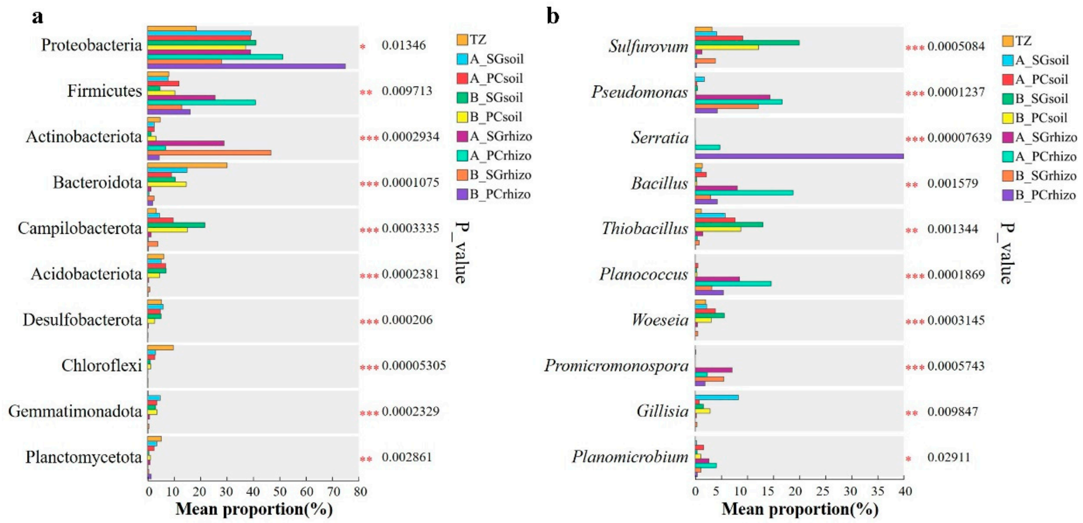

Figure S4 Relative abundance differences of bacterial communities in all soil samples based on the Kruskal–Wallis test, (a) phylum level, (b) genus level. TZ, tidal zone; A, *S. glauca* dominant area; B, *P. communis* dominant area; SG, *S. glauca*; PC, *P. communis*; soil, peri-root soil sample; rhizo, rhizosphere sample.
